# A rare case of *Stenotrophomonas maltophilia* endogenous endophthalmitis in pediatric age group

**DOI:** 10.1186/s12348-024-00431-z

**Published:** 2024-12-27

**Authors:** Tanya Jain, Alankrita Muralidhar, Arpan Gandhi

**Affiliations:** 1 Vitreoretina and Uvea Services, Dr Shroff’s Charity Eye Hospital, New Delhi, India; 2 Vitreoretina Services, Vivekananda Netralaya, Dehradun, India; 3 Laboratory Services, Dr. Shroff’s Charity Eye Hospital, New Delhi, India

**Keywords:** Endogenous endophthalmitis (EE), *Stenotrophomonas maltophilia*, Paediatric endogenous endophthalmitis, Endophthalmitis

## Abstract

**Background:**

We report a unique case of *Stenotrophomonas maltophilia*-related pediatric endogenous endophthalmitis.

**Case presentation:**

A 10-year-old male presented with redness and loss of vision in his right eye for two weeks. Clinical examination and ultrasound features were suggestive of endophthalmitis, most likely endogenous due to the absence of a history of trauma or intraocular intervention. Following vitrectomy and appropriate intravitreal antibiotics, the vision improved to 20/80. Vitreous culture revealed a gram-negative bacillus *Stenotrophomonas maltophilia*.

**Conclusion:**

Timely recognition of this pathogen and management as per antibiotic sensitivity can help salvage functional vision in this condition.

## Background

*Stenotrophomonas maltophilia* is a gram-negative, multidrug-resistant opportunistic pathogen that is responsible for nosocomial and community-acquired infections, especially in immunocompromised hosts [1]. Several case series document post-traumatic, post-surgical and post-intravitreal endophthalmitis (including cluster infections) caused by *S. maltophilia* [2–5]. However, endogenous endophthalmitis has been a rarely described entity, with seven documented cases in the literature, all of whom were adults [6–9]. Here we describe the rare case of a 10-year-old male who presented with endogenous endophthalmitis due to this pathogen.

## Case presentation

A 10-year-old male presented to the outpatient department of our hospital with complaints of loss of vision, redness, watering, and irritation in the right eye for 14 days. He was diagnosed elsewhere with a case of anterior uveitis for which he was started on hourly 1% prednisolone acetate and twice daily 2% homatropine eyedrops. Upon inquiry, he gave a history of fever 1 month ago along with a history of ear discharge in the left ear about 2 weeks before his eye symptoms started which resolved spontaneously. There was no history of ocular trauma or any previous ocular intervention (medical or surgical). On general physical examination, the child seemed under nourished, with thin and fragile hair. Upon further examination, the child’s weight (22 kg) and height (125 cm) fell below the 2nd standard deviation of normal according to the WHO (World Health Organization) weight for age and height for age charts [10].

The best corrected visual acuity was hand movements (HM) in the right and 20/20, N6 in the left eye. Intraocular pressure was recorded as 4 mm of mercury in the right eye and 12 mm hg in the left eye.

A slit lamp examination of the right eye revealed circumcorneal conjunctival congestion, a few keratic precipitates over the corneal endothelium with cells (grade 1), mild flare, and a streak hypopyon in the anterior chamber. The pupil was 5 mm dilated with posterior synechiae, a clear lens with dense vitreous exudates adherent to the posterior lenticular capsule was noted. There was no view of the fundus. The left eye examination was within normal limits.

**Fig. 1:**
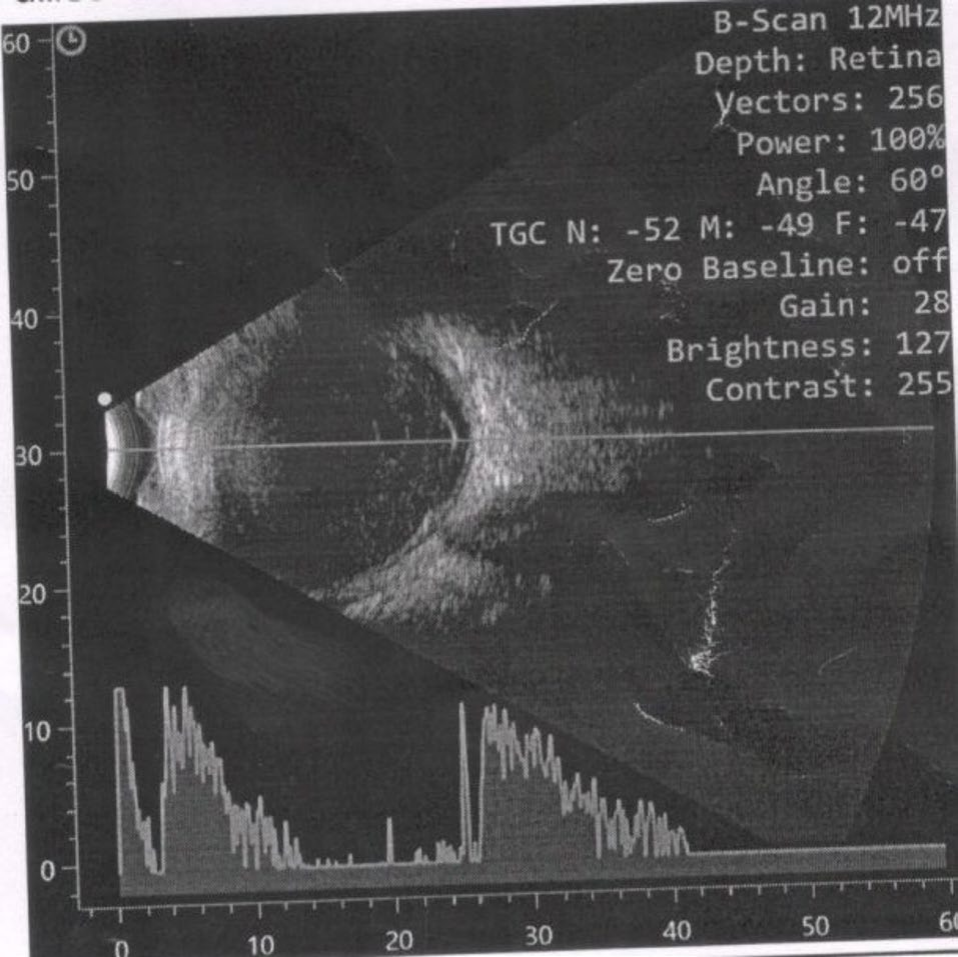
B scan ultrasonography at presentation: Shows a large number of low-to-medium reflective dots, clumps, and membranous echoes in the vitreous with choroidal thickening and an attached retina

B scan ultrasonography of the right eye revealed a large number of low-to-medium reflective dots, clumps, and membranous echoes in the vitreous with choroidal thickening and an attached retina (Fig. 1).

**Fig. 2:**
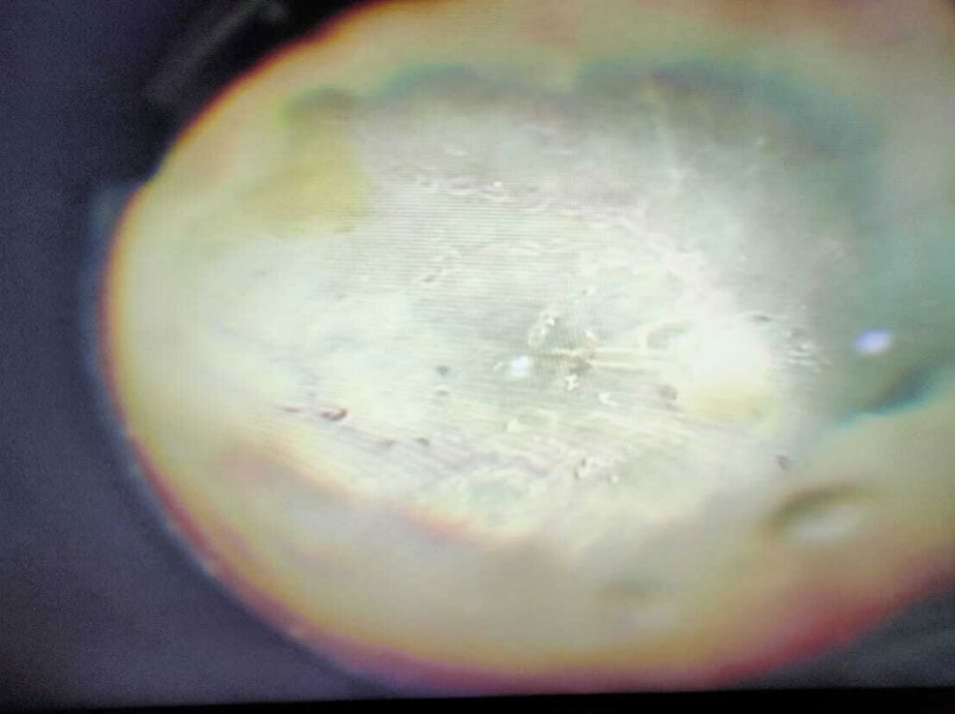
Intra-operative fundus photograph: Pale necrotic retina with extensive perivascular exudation seen after lensectomy and clearing of dense central exudates

After taking a written, valid informed consent, and obtaining fitness for anesthesia, the patient underwent a right eye lensectomy with a core vitrectomy with intraocular antibiotics under general anesthesia on the same day on an emergency basis. Twenty-three-gauge pars plana vitrectomy was performed. Before the infusion fluid was turned on, an undiluted vitreous sample was sent for a microbiological exam which included gram stain, 10% KOH (potassium hydroxide) mount as well as plating on culture media (Blood agar, Chocolate agar, Sabouroud’s dextrose agar). Despite a clear lens, a lensectomy was performed due to the presence of dense exudates stuck to the posterior lens capsule and poor pupillary dilation which made it difficult to remove these exudates without causing lens injury. Moreover, lensectomy facilitated safe and thorough vitrectomy which was crucial for managing the fulminant endophthalmitis. Following lensectomy and clearing of the dense central vitreous exudates, a pale necrotic retina with extensive perivascular exudation was noted (Fig. 2).

**Fig. 3:**
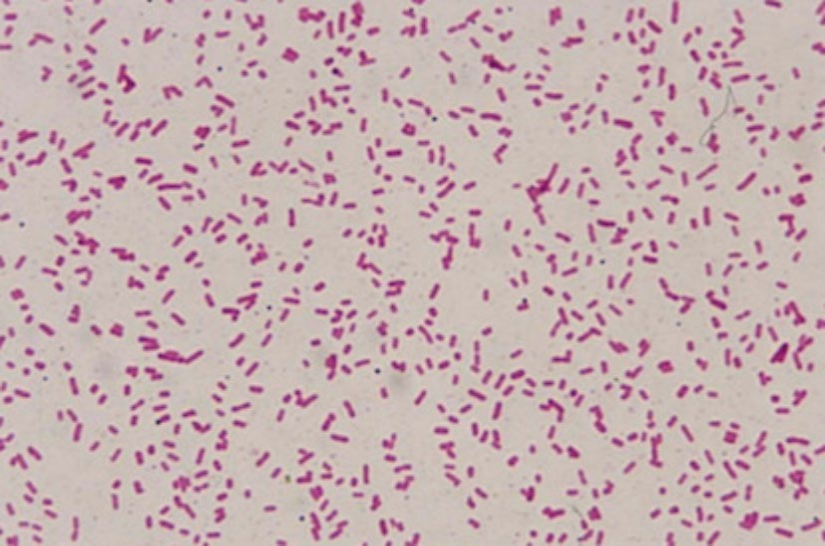
Gram’s stain of the teased growth from culture plate of vitreous exudate sample at 100x Magnification: Gram negative bacilli were noted

**Fig. 4:**
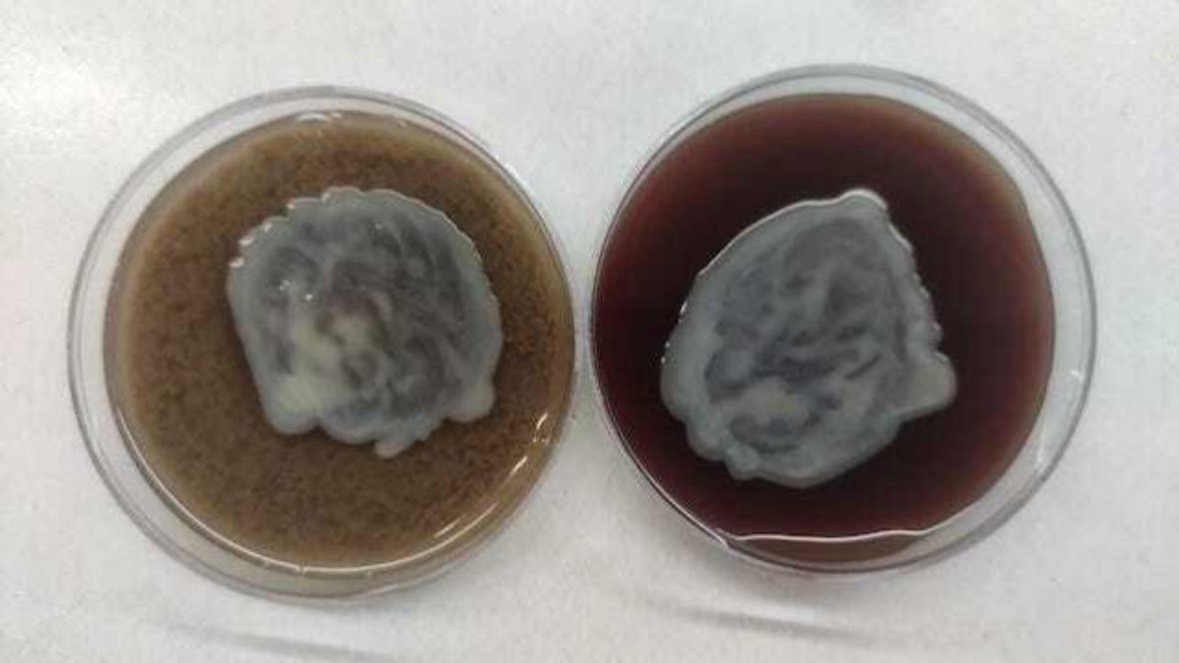
Bacterial culture plates showing growth of *Stenotrophomonas maltophilia* on blood agar (right) and chocolate agar (left). Colonies on blood agar were non-hemolytic, with a faint lavender color and an ammonia odor. They formed small colonies after 24 h at 37 °C, and then formed smooth, convex colonies about 3 mm in diameter after 48 h

Posterior vitreous detachment was not attempted, and core vitrectomy was completed. All ports were sutured with 7 − 0 vicryl, and intravitreal Vancomycin(1 mg/0.1 ml), ceftazidime(2.25 mg/0.1 ml), and dexamethasone(0.4 mg/0.1 ml) were injected at the end of the procedure. Postoperatively, the child was started on intravenous cefotaxime (500 mg 12 hourly), intravenous gentamicin (50 mg 8 hourly), oral prednisolone 20 mg once a day as well as topical prednisolone acetate hourly, atropine 4 hourly, and moxifloxacin hourly. On postoperative day 1, significant growth was observed on both blood and chocolate agar. Gram staining of the cultured bacteria revealed the presence of gram-negative bacilli (Figs. 3 and 4). Species identification and antibiotic susceptibility testing were pending.

Urine and blood cultures showed no growth. Due to the fulminant clinical presentation, *Pseudomonas sp*. was suspected, and the child was administered intravitreal Imipenem (100µgm/ 0.1 ml) with dexamethasone(0.4 mg/0.1 ml) on postoperative day 1 due to the high risk of rapid clinical deterioration while awaiting identification and antibiotic sensitivity results. On postoperative day 3 bacterial isolate of *Stenotrophomonas maltophilia* was identified from culture using the Vitek microbiology analyzer followed by confirmatory biochemical tests which showed positive Catalase and negative Oxidase tests.

**Fig. 5:**
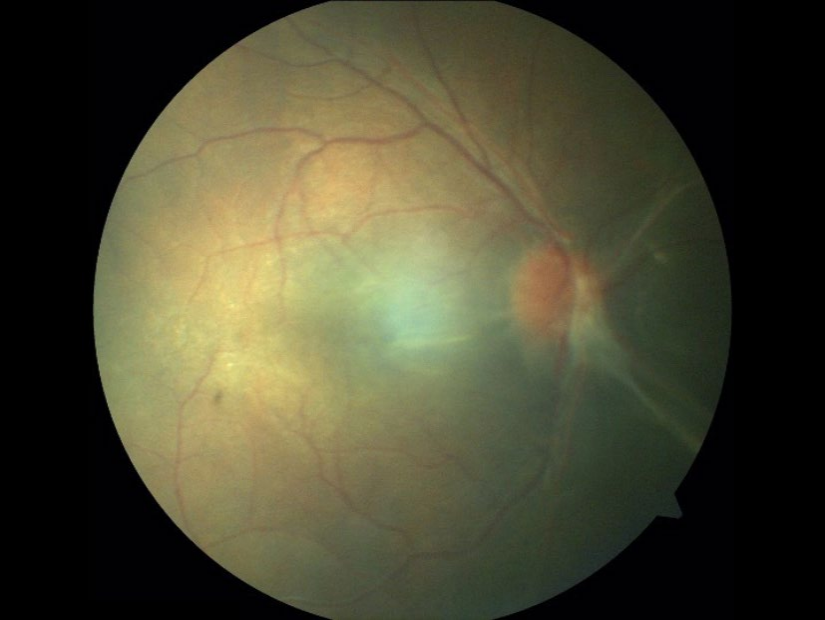
Colour Fundus photograph at 6 month follow up visit: Shows a resolving disc edema with fibrous bands causing traction over the macula and nasal retina with old sheathing on peripheral vessels

Antibiotic susceptibility testing performed using the Kirby Bauer disc diffusion method showed sensitivity to fluoroquinolones (ciprofloxacin, levofloxacin, ofloxacin, gatifloxacin), chloramphenicol, gentamicin, and resistance to both ceftazidime and imipenem. Intravitreal moxifloxacin (0.05 ml from topical moxifloxacin 0.5%) and dexamethasone(0.4 mg/0.1 ml) was injected on the same day and was repeated every alternate day till fundus examination showed resolution of perivascular exudation. A total of five doses of intravitreal moxifloxacin and dexamethasone were administered at 48-hour intervals. Intravenous antibiotics (cefotaxime 500 mg 12 hourly and gentamicin 50 mg 8 hourly) were continued till post-operative day 7 and subsequently, the child was given oral ciprofloxacin 250 mg twice a day for a week. Oral prednisolone was tapered weekly and stopped. At 1 month post operatively, the best corrected distance visual acuity was 20/80 with + 14.00 D of aphakic correction with a near visual acuity of N-10 with + 3.00 D addition. Fundus examination showed a resolving disc edema with fibrous bands causing traction over the macula and nasal retina with old sheathing on peripheral vessels. At the 6-month final follow-up, the best corrected visual acuity was maintained at 20/80(N-10) with stable fundus findings (Fig. 4). The child was using aphakic correction and was planned for a scleral fixated intraocular lens placement but was lost to follow-up (Figure 5).

## Discussion

*Stenotrophomonas maltophilia* has been widely reported to cause post-surgical and post-intravitreal endophthalmitis [2–5]. Endogenous endophthalmitis (EE) secondary to this organism has been rarely reported, with the most common age group being from 22 to 35 years (mean age 26.5 years) [6–9]. To the best of our knowledge, there have been no cases of *S. maltophilia* endogenous endophthalmitis reported in the pediatric age group. Pediatric endogenous endophthalmitis has been reported to have a relatively high incidence (28.9%) in the Indian subtext, with the likely cause being immunocompromise secondary to malnutrition in this part of the world [11]. EE is usually associated with systemic symptoms such as fever, malaise and underlying risk factors such as recent hospitalization, diabetes mellitus, urinary tract infection, immunosuppression (especially associated with underlying malignancy, neutropenia, and HIV (human immunodeficiency virus)), and indwelling catheters [12]. On inquiry, we elicited a history of febrile illness approximately 1 month prior along with a history of ear discharge about 2 weeks before the onset of ocular symptoms. With the absence of any surgical intervention, trauma, signs of any disruption of ocular structure integrity or intra-ocular foreign body, a diagnosis of EE was made. The low socio-economic status and malnutrition may have further contributed to the development of the disease. The lack of systemic features in paediatric EE in the Indian setting has been attributed to the high prevalence of protein energy malnutrition-related immunocompromise [11]. The antibiotic susceptibility in our case was similar to other reported cases with fluoroquinolone susceptibility and resistance to carbapenems and cephalosporins(ceftazidime) [2, 4, 5]. The visual outcome in our case was like existing reported cases where six out of seven cases had a visual outcome better than 6/60, with only one having light perception vision due to retinal detachment following vitrectomy, to which the patient did not consent for further intervention [6–9].

## Conclusion

*Stenotrophomonas maltophilia* can rarely cause endogenous endophthalmitis in the pediatric age group especially in the Indian setting where protein energy malnutrition can cause an immunocompromised state. Timely diagnosis with high clinical suspicion is of paramount importance due to the fulminant nature of infection caused by this organism.Early surgical intervention, and medical management as per culture and antibiotic susceptibility can eradicate ocular infection and salvage functional vision.

## Data Availability

No datasets were generated or analysed during the current study.

## References

[1] Brooke JS (2012) *Stenotrophomonas maltophilia*: an Emerging Global Opportunistic Pathogen. Clin Microbiol Rev 25(1):2–41 22232370 10.1128/CMR.00019-11 PMC3255966

[2] Chen K-J, Wang N-K, Sun M-H, Chen T-L, Lai C-C, Wu W-C et al (2010) Endophthalmitis caused by *Stenotrophomonas maltophilia*. Ophthalmic Surg Lasers Imaging 41(5):e555–e556 20873692 10.3928/15428877-20100910-03

[3] Chang JS, Flynn HW Jr, Miller D, Smiddy WE (2013) *Stenotrophomonas maltophilia* endophthalmitis following cataract surgery: clinical and microbiological results. Clin Ophthalmol 7:771–777 23620659 10.2147/OPTH.S39608 PMC3633579

[4] Ji Y, Jiang C, Ji J, Luo Y, Jiang Y, Lu Y (2015) Post-cataract endophthalmitis caused by multidrug-resistant *Stenotrophomonas maltophilia*: clinical features and risk factors. BMC Ophthalmol 15(1):14 25618260 10.1186/1471-2415-15-14 PMC4320429

[5] Agarwal AK, Aggarwal K, Samanta R, Angrup A, Biswal M, Ray P et al (2019) Cluster endophthalmitis due to *Stenotrophomonas maltophilia* following intravitreal bevacizumab: outcomes of patients from North India. Br J Ophthalmol 103(9):1278–1283 30420442 10.1136/bjophthalmol-2018-313131

[6] Das T, Deshmukh HS, Mathai A, Reddy AK (2009) *Stenotrophomonas maltophilia* endogenous endophthalmitis: clinical presentation, sensitivity spectrum and management. J Med Microbiol 58:837–838 19429764 10.1099/jmm.0.009431-0

[7] Chhablani J, Sudhalkar A, Jindal A, Das T, Motukupally SR, Sharma S et al (2014) *Stenotrophomonas maltophilia* endogenous endophthalmitis: clinical presentation, antibiotic susceptibility, and outcomes. Clin Ophthalmol 8:1523–1526 25170244 10.2147/OPTH.S67396 PMC4144939

[8] Suhan D, Kolavali RR, Kelgaonkar A (2021) Sieve-like preretinal exudates in *Stenotrophomonas maltophilia* endogenous endophthalmitis. BMJ Case Rep 14(7):244–392 10.1136/bcr-2021-244392 PMC8314735 34312142

[9] Bilgic A, Sudhalkar A, Gonzalez-Cortes JH, March de Ribot F, Yogi R, Kodjikian L et al (2021) Endogenous endophthalmitis in the setting of covid-19 infection: a Case Series. Retina 41(8):1709–1714 33734193 10.1097/IAE.0000000000003168

[10] World Health Organization Growth reference data for 5 to 19 years. [Website]. https://www.who.int/tools/growth-reference-data-for-5to19-years/indicators. Accessed 28 August 2024

[11] Maitray A, Rishi E, Rishi P, Gopal L, Bhende P, Ray R et al (2019) Endogenous endophthalmitis in children and adolescents: Case series and literature review. Indian J Ophthalmol 67(6):795–800 31124489 10.4103/ijo.IJO_710_18 PMC6552604

[12] Connell PP, O’Neill EC, Fabinyi D, Islam FMA, Buttery R, McCombe M et al (2011) Endogenous endophthalmitis: 10-year experience at a tertiary referral centre. EYE 25(1):66–72 20966972 10.1038/eye.2010.145 PMC3144637

